# A Multiple-Classifier Framework for Parkinson's Disease Detection Based on Various Vocal Tests

**DOI:** 10.1155/2016/6837498

**Published:** 2016-04-12

**Authors:** Mahnaz Behroozi, Ashkan Sami

**Affiliations:** Department of CSE and IT, School of Electrical Engineering and Computer Science, Shiraz University, Shiraz 71348-51154, Iran

## Abstract

Recently, speech pattern analysis applications in building predictive telediagnosis and telemonitoring models for diagnosing Parkinson's disease (PD) have attracted many researchers. For this purpose, several datasets of voice samples exist; the UCI dataset named “*Parkinson Speech Dataset with Multiple Types of Sound Recordings*” has a variety of vocal tests, which include sustained vowels, words, numbers, and short sentences compiled from a set of speaking exercises for healthy and people with Parkinson's disease (PWP). Some researchers claim that summarizing the multiple recordings of each subject with the central tendency and dispersion metrics is an efficient strategy in building a predictive model for PD. However, they have overlooked the point that a PD patient may show more difficulty in pronouncing certain terms than the other terms. Thus, summarizing the vocal tests may lead into loss of valuable information. In order to address this issue, the classification setting must take* what* has been said into account. As a solution, we introduced a new framework that applies an independent classifier for each vocal test. The final classification result would be a majority vote from all of the classifiers. When our methodology comes with filter-based feature selection, it enhances classification accuracy up to* 15%*.

## 1. Introduction

Parkinson's disease was first introduced in 1817 by Doctor James Parkinson as “shaking palsy” [1]. It is the second common neurological disease coming afterwards Alzheimer and is mostly common among elders [2, 3]. PD is a kind of progressive disease in which an area of brain becomes damaged over the years. It causes various signs and symptoms. From one perspective, these signs and symptoms can be grouped into two major categories: motor symptoms and nonmotor symptoms. Motor symptoms are those that affect movement and muscles and nonmotor symptoms include neurobehavioral and cognitive problems, sleep problems, sensory problems, and autonomic neuropathy (dysautonomia) [4].

Speech disturbance is one of the most common motor problems of PD [4]. Research has shown that about 90% of PWP are affected with motor problems, especially speech impairment [5, 6]. In addition to the prevalence of vocal impairments in PD patients, gathering speech samples and doing signal processing of their voice has low cost and it is appropriate for telemonitoring and telediagnosis systems [7, 8]. Therefore, PD diagnosis from speech impairments is becoming more widespread.

In Parkinsonism patients, speech disorders result from neurologic impairments which are associated with weakness, slowness, or incoordination of the muscles used to produce speech [9, 10]. Speech disturbance usually occurs in the following forms:* hypophonia*, which is soft speech that results from weakness in the vocal musculature,* monotonic speech*, which deals with speech quality in the cases that are soft, hoarse, and monotonous, and* festination speech*, which is when the speech becomes excessively rapid, soft, breathy, and poorly intelligible [4].

Many approaches have been proposed in order to find the severity of each speech impairment sign. There are two types of the best known vocal tests for this purpose:* sustained phonation* [11, 12] and* running speech* [12] tests. In sustained phonation, the patient is asked to say a single vowel, while holding its pitch as constant and long as possible. In running speech, the patient says a standard sentence which includes representative linguistic units that can show possible impairment signs of vocal disorder. The main focus of this research is on the latter problem statement. Previous researches had two main flaws: (a) all the voice samples were classified by a single classifier; (b) the vocal samples of each subject were summarized with the help of statistical metrics irrespective of discriminating ability of each vocal test.

Since most studies in the area of PD detections based on speech are done on datasets gathered on just one or a few types of vocal tests, we have brought our attention to a dataset with multiple sound recordings. The main contributions of this study are twofold: (1) to suggest a new distinctive classification framework which proposes to apply a unique classifier to vocal samples of* each type*, for example, have a classifier just for vowel “a,” rather than applying a single classifier for all vocals and (2) to present which vocal tests are more representative and to indirectly omit less discriminating vocal tests by embedding majority voting in our proposed method.

The rest of the paper is organized as follows.  Section 2 reviews previous studies of this domain. In Section 3, a brief description of the dataset, evaluation metrics, and the proposed method can be found.  Section 4 demonstrates the results of this work and, finally, Section 5 presents the conclusion of this study.

## 2. Related Work

In recent years the detection of vocal disorders with the help of machine learning turned into a hot topic. Various research papers have attempted to solve this problem by considering acoustic measurements of dysphonia as effective features to distinguish normal (control) from disordered cases [7, 8, 13, 14]. Studies in this field can be categorized into two main groups: (1) those that attempt to find the most effective vocal features and produce new datasets [8, 13, 15] and (2) those that try to find more effective features from existing datasets and concentrate on enhancing classification accuracy [14, 16–26].

Some studies focused on how to produce new datasets based on their research findings. Little et al. in [8] aimed to analyze the effectiveness of nonstandard measurements. Their work led to the introduction of a new dysphonia measurement named as PPE (pitch period entropy). In their study, they had collected sustained vowel “a” phonations from 31 subjects of which 23 were PD patients and they reached the classification accuracy of 91.4%. In [13], Sakar et al. presented a dataset of 40 subjects including 20 PD. Each individual was trained to say a set of 26 distinct disorder representative terms consisting of sustained vowels, words, numbers, and short sentences. This dataset is the focus of current work. They applied* summarized leave-one-out* (s-LOO) validation technique in which all the voice samples of each individual will be summarized using central tendency and dispersion metrics such as median, mean, standard deviation, trimmed mean, interquartile range, and mean absolute deviation. Their approach obtained 77.5% of classification accuracy. Tsanas et al. in [15] focused on monitoring the PD progression with the help of extracted features using signal processing techniques applied on a huge dataset of about 6000 voice samples from 42 patients with early-stage PD. They have attempted to estimate the unified Parkinson's disease rating scale (UPDRS) using linear and nonlinear regression. Their results show the accuracy of about 7.5-point difference from clinical UPDRS estimations. These three datasets are the main publicly available datasets of PD speech-based area of study.

Other studies tried to improve the PD detection rate using the existing datasets. For instance, Tsanas et al. in [14] computed 132 dysphonia new measurements using an existing dataset consisting of 263 vowels “ahh…” phonations from 43 cases by applying feature selection techniques. They obtained 99% overall classification accuracy. In another work, Sakar and Kursun [16] tried to assess the relevance and correlation between the features and PD score by applying mutual information-based selection algorithm with permutation test and feed the data with selected features ranked based on maximum-relevance-minimum-redundancy (mRMR) into an SVM classifier. They used leave-one-subject-out (LOSO) as the cross validation technique of their model in order to avoid bias. In LOSO validation scheme, all the voice samples of an individual which is the candidate of being the testing sample will be left out from the rest of the data. Their approach gained 92.75% classification accuracy [8]. Shahbaba and Neal [17] presented a nonlinear model based on Dirichlet mixtures and obtained the classification accuracy of 87.7%. Das [18] conducted a comparative study of neural networks (NN), DMneural, regression, and decision trees for PD diagnosis; their study resulted in classification performance of 92.9% based on NN. Guo et al. [19] applied a combination of genetic programming and the expectation maximization (EM) and obtained a classification accuracy of 93.1%. Luukka [20] proposed a method that used fuzzy entropy measures and similarity classifier and resulted in the mean accuracy of 85.03%. Li et al. [21] introduced a fuzzy-based nonlinear transformation approach combined with SVM; their best classification accuracy was 93.47%. Ozcift and Gulten [22] proposed classifier ensemble construction with a rotation forest approach which got classification accuracy of 87.13%. Åström and Koker [23] achieved the classification accuracy of 91.2% by using a parallel neural network model. Polat [24] applied the fuzzy* C*-means clustering feature weighting together with the* k*-nearest neighbor classifier; their best obtained classification accuracy was 97.93%. Chen et al. [25] proposed a model which combined PCA and the fuzzy* k*-nearest neighbor method; their classification approach achieved an accuracy of 96.07%. Zuo et al. [26] used a diagnosis model based on particle swarm optimization (PSO) to strengthen the fuzzy* k*-nearest neighbor classifier which resulted in mean classification accuracy of 97.47%.

In most of the studies, SVM was used as the base classifier to distinguish healthy subjects from PWP [8, 14, 27] and the success of the diagnostic system is measured with ROC curves, AUC, and reporting True Positive and False Positive rates [28].

The speech datasets used in the field of PD diagnosis consist of multiple speech recordings per subject [29]. These datasets can be grouped into two categories: (1) those that contain the repetition of one term and (2) those that consist of* different* vocal terms. The majority of datasets go to the first category. Hence, most of the studies on PD diagnosis are conducted on these datasets [14, 16–26]; however, none of them could obtain 100% classification accuracy. The most popular and available datasets of this type are “*Parkinson's Data Set*” [7] and “*Parkinson's Telemonitoring Data Set*” [15], both accessible from* UCI Machine Learning Repository*. The only dataset of the second category that is available in the form of processed data matrix was produced by Sakar et al. [13]. Less research has been conducted on this type of datasets; also, corresponding classification accuracies are not promising up to this time. The aim of this study is to show that this type of data collection can lead to high PD detection rates just by altering the classification strategy.

## 3. Materials and Methods

### 3.1. Data

In this work we used* Parkinson Speech Dataset with Multiple Types of Sound Recordings* [13], which is available on the University of California, Irvine (UCI) machine learning dataset repository website. This dataset consists of 40 subjects, including 20 PD patients and 20 control subjects. For each subject, 26 different sound recordings have been gathered, consisting of three sustained vowels, numbers one through 10, nine words, and four short sentences. There are 26 features extracted from these recordings.  Table 1 lists the features gathered in Sakar et al.'s work and their corresponding groups (see [13] for more details).

### 3.2. Overview of the Proposed Method

**Figure 1:**
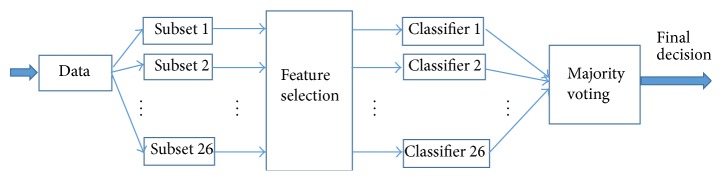
An illustration of the proposed method.

The aim of this paper is to propose a classification framework which focuses on the discriminating values of each vocal test. Unlike the conventional LOSO cross validation technique and s-LOO, the proposed methodology considers that all the recordings in the setting of the dataset are not necessarily discriminating and not every PD patient demonstrates distinguishable vocal disorders in all vocal tests. The overall view of the proposed method can be seen in Figure 1.

The four steps of the proposed methodology are (1) separating the dataset based on the types of sounds recorded, (2) applying feature selection with Pearson Correlation Coefficient, we called the two approaches we took as follows: (a)* Multiple-Classifier with Feature Selection* (MCFS) and (b)* Adjusted Multiple-Classifier with Feature Selection* (A-MCFS) which will be introduced shortly, (3) applying a classifier on each subset, and finally (4) fusing the results of all classifiers to obtain the final decision by means of majority voting.

(*1) Data Separation*. Each subset of the dataset includes all the vocal tests of the same type; for instance, all the recorded vowel sounds of type “a” go to the first subset and those of “o” go to the second subset. Thus, considering the present dataset, there are 26 subsets, each containing 40 samples. Separation has been done since the mixture of all the voice samples of an individual diminishes the discriminating effect of more descriptive tests and it will affect the classification results negatively.

(*2) Pearson Correlation Coefficient Feature Selection*. For feature selection phase, a filter-based feature selection technique based on Pearson Correlation Coefficient [30–32] was used to find highly correlated features to the class label. A more precise definition of this feature selection is as follows: suppose that each feature consists of {*x*_1_, *x*_2_,…, *x*_*n*_} values for samples 1 through *n* (in this study *n* is equal to 40) in vector **X** and the corresponding class labels are {*y*_1_, *y*_2_,…, *y*_*n*_} stored in vector **Y**. So the Pearson Correlation Coefficient of each feature can be calculated as

(1) $$\begin{array}{c} r=r_{XY}=\frac{\sum_{i=1}^{n}\left(x_{i}-\overset{-}{x}\right)\left(y_{i}-\overset{-}{y}\right)}{\sqrt{\sum_{i=1}^{n}{\left(x_{i}-\overset{-}{x}\right)}^{2}}\sqrt{\sum_{i=1}^{n}{\left(y_{i}-\overset{-}{y}\right)}^{2}}}, \end{array}$$

where $\overset{-}{x}=(1/n)\sum_{i=1}^{n}x_{i}$ and similarly $\overset{-}{y}=(1/n)\sum_{i=1}^{n}y_{i}$. This equation gives a value between −1 and +1, where +1 is maximum positive correlation, 0 is no correlation, and −1 is the strongest negative correlation.

**Figure 2:**
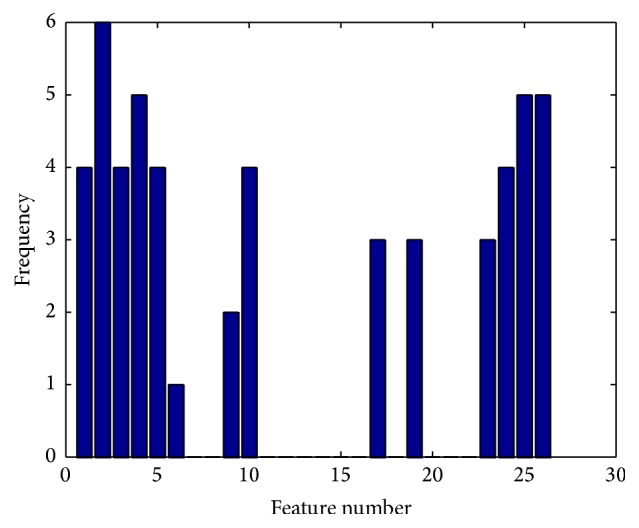
Frequency of each selected feature as relevant feature of a vocal test.

The *p* values were calculated using Student's *t*-distribution for a transformation of the correlation. Those features in the correlation coefficient matrix with *p* values less than 0.05 were selected.

(*3) MCFS and A-MCFS*. When the Pearson Correlation Coefficient feature selection is applied, some vocal tests may remain with no relevant features. We call those vocal tests as* unsuccessful vocal tests*. Two approaches for dealing with those unsuccessful vocal tests are taken in this study. The first is the MCFS approach; the vocal tests are used in the analysis only based on the prevalent features of other vocal tests.  Table 2 shows each vocal test and its corresponding correlated features after applying feature selection and Figure 2 shows the frequency of each feature. Features 2 and 4 with frequency of six and five were, respectively, the most frequent selected features. The third most frequent was shared by features 25 and 26 with frequency of four. The most four frequent features were used in MCFS as selected features for unsuccessful vocal tests. The other methodology, A-MCFS, is to simply omit unsuccessful vocal tests.

(*4) Classification and Majority Voting*. After doing feature selection on each subset, for each of them, a classifier is built. Since we have 26 vocal tests, 26 classifiers are built. Each of these classifiers will predict the class label of its own subset.* Leave-one-out* cross validation technique was used for all of these classifiers. Since each subject has only one record in each subset, we do not have to worry about how to treat each subject for doing cross validation as it was the case in LOSO or previous approaches.

The majority vote of classifiers decides which class the person belongs to. Each classifier votes whether the subject has PD or not. Then, the subject whom the majority of the classifiers have voted to be a PD patient will be labeled as “1” showing the presence of PD and “0” otherwise.

### 3.3. Evaluation Metrics

The evaluation metrics used to show the effectiveness of the proposed methodology are accuracy, sensitivity, specificity, and Matthew's correlation coefficient score (MCC). The definitions of these metrics are as follows:

(2) $$\begin{array}{c} accuracy=\frac{TP+TN}{TP+TN+FP+FN}, \end{array}$$

where TP (True Positive) is the number of PD patients who are correctly classified as Parkinsonism patients by the model, TN (True Negative) is the number of control subjects who are labeled as healthy by the model, FN (False Negative) is the number of patients that the model falsely labeled them as healthy, and finally FP (False Positive) is the number of healthy cases who are incorrectly labeled as having PD by the classifier. It simply shows that the accuracy is the ratio of the correctly classified samples to the total number of instances:

(3) sensitivity=TPTP+FNspecificity=TNTN+FP.

A well-known metric in machine learning which can be used for evaluating the quality of a binary classifier is MCC. This metric is reliable since it takes TP, TN, FN, and FP into account and this makes it stable even if classes are of very different sizes. Actually, MCC is a correlation coefficient between observed (actual) labels of the samples and those predicted by the binary classifier:

(4) MCC=TP×TN−FP×FNTP+FPTP+FNTN+FPTN+FN.

This equation returns a value between −1 and +1. A coefficient of +1 shows a perfect prediction, 0 represents the fact that the classifier is not better than random guessing, and finally −1 indicates a complete disagreement between the actual values and the predicted ones.

## 4. Results and Discussions

After separating the data into subsets, the *z*-score normalization process was applied on each subset. In other words, after transformation, mean is equal to zero and standard deviation changes to one. Then the proposed framework was applied on the refined data.

Four classifiers including* k-*NN, SVM, discriminant analysis, and Naïve Bayes were applied to the preprocessed data. Distance metric used for the* k*-NN classifier was Euclidean distance and *k* with values of 1, 3, 5, and 7 was used. SVM classifier was applied using linear and radial basis kernels (RBF) with scaling factor (sigma) of 3 and penalty parameter (*C*) of 1.  Table 3 includes the accuracy, sensitivity, specificity, and MCC obtained from applying mentioned classifiers under LOSO, s-LOO, and the proposed frameworks. The results reveal that* k*-NN classifier performance is almost analogous to random guessing when it is used with LOSO cross validation technique. Besides that, s-LOO could not perform much better than LOSO when it comes to* k*-NN since its best overall accuracy and MCC are 65.00% and 0.3062, respectively. Results show that A-MCFS outperforms s-LOO's best result with overall accuracy of 77.50%, which is a** 12.5%** improvement and MCC of 0.5507.

When *k* is 1, 3, and 5, MCFS results are better than s-LOO at least for 5% and at most 12.5%, but its accuracy is 2.5% lower than s-LOO when *k* is 7.

Sensitivity is another important factor, especially in biomedical sciences, which should be investigated closely in the results. As the results show, A-MCFS also has improved the sensitivity up to 80.00% and its lowest sensitivity (70.00%) is still better than that of LOSO and s-LOO when* k-*NN is used.* k*-NN achieved its best results when A-MCFS was applied; besides this, LOSO and s-LOO could not reach MCFS's results except for* k* = 7.

In addition, the best classification accuracy obtained by applying A-MCFS is 87.5% which is a 10% accuracy enhancement in comparison to the best accuracy obtained by s-LOO.

**Figure 3:**
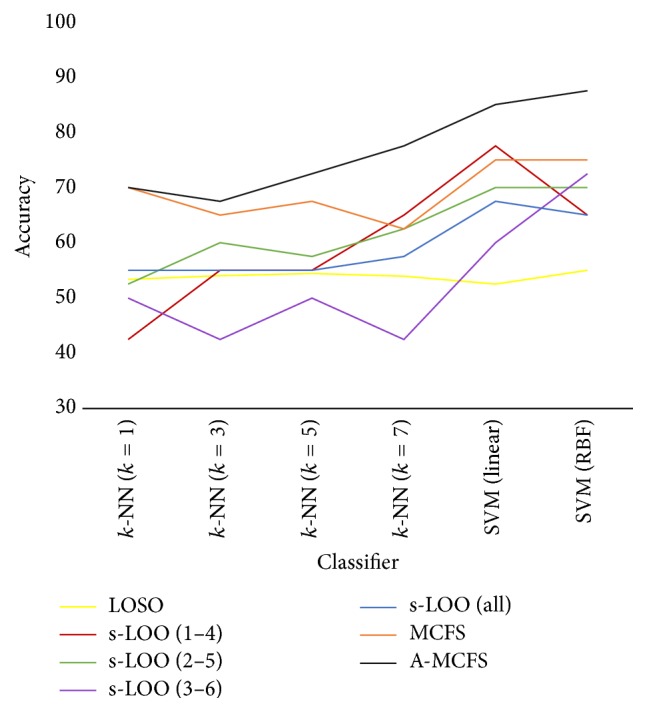
Obtained accuracies based on the reported results in Table 3.

Figure 3 gives a better demonstration of classification accuracies obtained by different methods.

In order to examine the correctness of our approach toward finding less discriminating vocal tests, we have reported the classification accuracy of each vocal test prior to the majority voting phase. The results are shown in Table 4. Comparing the results shown in Tables 2 and 4 reveals that the features which were excluded in A-MCFS are those that achieved a mean accuracy of below 55%. This shows the reason of the superiority of A-MCFS over MCFS. As a result, a closer investigation toward finding more effective vocal tests is necessary.

## 5. Conclusion

PWP detection based on vocal samples has been an attractive area of research. Finding a solution toward discriminating PD patients from the healthy people based on different vocal tests had been less accurate since all the vocal terms were treated by a single classifier. The proposed method treated each vocal test separately and used majority voting to resolve any potential confusion. Obtained results from this research showed that more accurate PD detection based on multiple vocal tests is achievable.

Another important result, achieved from this study, was that the discriminating ability of all the vocal terms is not the same, even some of those vocal terms that have been considered to be discriminating in the literature, such as vowel “a,” failed to be successful. As a result, our study may encourage other researchers to conduct further studies on different vocal terms from the proposed perspective.

As the future work, we plan to devise a laboratory setting to collect data from PWP and healthy subjects with several vocal tests from various languages and extend our results to other languages.

## Figures and Tables

**Table 1:** Time-frequency based features presented in Parkinson speech dataset with multiple types of sound recordings [13].

Feature number	Feature name	Group
1	Jitter (local)	*Frequency parameter*
2	Jitter (local, absolute)	
3	Jitter (rap)	
4	Jitter (ppq5)	
5	Jitter (ddp)	

6	Number of pulses	*Pulse parameters *
7	Number of periods	
8	Mean period	
9	Standard deviation of period	

10	Shimmer (local)	*Amplitude parameters*
11	Shimmer (local, dB)	
12	Shimmer (apq3)	
13	Shimmer (apq5)	
14	Shimmer (apq11)	
15	Shimmer (dda)	

16	Fraction of locally unvoiced frames	*Voicing parameters*
17	Number of voice breaks	
18	Degree of voice breaks	

19	Median pitch	*Pitch parameters*
20	Mean pitch	
21	Standard deviation	
22	Minimum pitch	
23	Maximum pitch	

24	Autocorrelation	*Harmonicity parameters*
25	Noise-to-harmonic	
26	Harmonic-to-noise	

**Table 2:** Each vocal test and its related features after applying filter-based feature selection.

ID	Vocal test	Related features
1	Vowel “a”	None
2	Vowel “o”	24
3	Vowel “u”	None
4	Number 1	1, 2, 3, 4, 5, 24
5	Number 2	2, 9, 10
6	Number 3	17, 19, 23, 25, 26
7	Number 4	1, 2, 3, 4, 5, 10
8	Number 5	24
9	Number 6	None
10	Number 7	None
11	Number 8	9
12	Number 9	26
13	Number 10	None
14	Short sentence 1	None
15	Short sentence 2	25, 26
16	Short sentence 3	4, 10, 25, 26
17	Short sentence 4	1, 2, 3, 4, 5, 10, 26
18	Word 1	2
19	Word 2	None
20	Word 3	17, 19, 23, 25
21	Word 4	None
22	Word 5	None
23	Word 6	None
24	Word 7	None
25	Word 8	1, 2, 3, 4, 5, 6, 17, 19, 23, 25
26	Word 9	24

**Table 3:** Results obtained from applying different methods and classifiers.

Classifier	Method	Accuracy (%)	Sensitivity (%)	Specificity (%)	MCC
*k*-NN (*k* = 1)	LOSO	53.37	49.62	57.12	0.0007
	s-LOO (1–4)	42.50	30.00	55.00	0.0015
	s-LOO (2–5)	52.50	45.00	60.00	0.0005
	s-LOO (3–6)	50.00	55.00	45.00	0.0000
	s-LOO (all)	55.00	55.00	55.00	0.1000
	MCFS	67.50	75.00	60.00	0.3549
	A-MCFS	**70.00**	**80.00**	**60.00**	**0.4082**

*k*-NN (*k* = 3)	LOSO	54.04	53.27	54.81	0.0008
	s-LOO (1–4)	55.00	45.00	65.00	0.1021
	s-LOO (2–5)	60.00	55.00	65.00	0.2010
	s-LOO (3–6)	42.50	55.00	30.00	0.0015
	s-LOO (all)	55.00	55.00	55.00	0.1000
	MCFS	65.00	60.00	70.00	0.3015
	A-MCFS	**67.50**	**75.00**	**60.00**	**0.3540**

*k*-NN (*k* = 5)	LOSO	54.42	53.65	55.19	0.0009
	s-LOO (1–4)	55.00	45.00	65.00	0.1201
	s-LOO (2–5)	57.50	65.00	50.00	0.1517
	s-LOO (3–6)	50.00	70.00	30.00	0.0000
	s-LOO (all)	55.00	70.00	40.00	0.1048
	MCFS	67.5	60.00	75.00	0.3540
	A-MCFS	**72.50**	**70.00**	**75.00**	**0.4506**

*k*-NN (*k* = 7)	LOSO	53.94	54.04	53.85	0.0008
	s-LOO (1–4)	65.00	55.00	75.00	0.3062
	s-LOO (2–5)	62.50	60.00	65.00	0.2503
	s-LOO (3–6)	42.50	65.00	20.00	0.0017
	s-LOO (all)	57.50	65.00	50.00	0.1517
	MCFS	62.5	65.00	60.00	0.2503
	A-MCFS	**77.50**	**80.00**	**75.00**	**0.5507**

SVM (linear kernel)	LOSO	52.50	52.50	52.50	0.0006
	s-LOO (1–4)	77.50	80.00	75.00	0.5507
	s-LOO (2–5)	70.00	80.00	60.00	0.4082
	s-LOO (3–6)	60.00	65.00	45.00	0.2000
	s-LOO (all)	67.50	70.00	65.00	0.3504
	MCFS	75.00	75.00	75.00	0.5000
	A-MCFS	**85.00**	**85.00**	**85.00**	**0.6000**

SVM (RBF kernel)	LOSO	55.00	60.00	50.00	0.1005
	s-LOO (1–4)	65.00	60.00	70.00	0.3015
	s-LOO (2–5)	70.00	70.00	70.00	0.4000
	s-LOO (3–6)	72.50	70.00	75.00	0.4506
	s-LOO (all)	65.00	70.00	60.00	0.3015
	MCFS	75.00	80.00	70.00	0.5025
	A-MCFS	**87.50**	**90.00**	**85.00**	**0.7509**

Naïve Bayes	MCFS	75.00	90.00	60.00	0.5241
	A-MCFS	**80.00**	**80.00**	**80.00**	**0.6000**

Discriminant analysis	MCFS	72.50	75.00	70.00	0.4506
	A-MCFS	**82.50**	**80.00**	**85.00**	**0.6508**

*Central tendency metrics used in s-LOO method*: 1: mean, 2: median, and 3: trimmed mean (25% removed).

*Dispersion metrics used in s-LOO method*: 4: standard deviation, 5: mean absolute deviation, and 6: interquartile range.

**Table 4:** Prediction ability of each vocal test, based on their obtained classification accuracy.

Vocal test ID	Classification accuracy (%)								
	*k*-NN (*k* = 1)	*k*-NN (*k* = 3)	*k*-NN (*k* = 5)	*k*-NN (*k* = 7)	SVM (linear kernel)	SVM (RBF kernel)	Naïve Bayes	DA	Mean accuracy ± standard deviation
1	42.5	35	27.5	27.5	47.5	25	52.5	37.5	**36.9 ± 10**
2	57.5	67.5	70	70	62.5	62.5	60	70	65 ± 5
3	40	42.5	60	55	27.5	50	50	47.5	**46.56 ± 10**
4	67.5	70	75	67.5	60	65	65	62.5	66.6 ± 4.6
5	67.5	57.5	60	65	67.5	67.5	67.5	65	64.7 ± 3.9
6	62.5	67.5	67.5	72.5	62.5	72.5	72.5	65	67.8 ± 4.3
7	52.5	60	57.5	55	67.5	67.5	70	50	60 ± 7.6
8	57.5	62.5	62.5	70	65	67.5	65	62.5	64 ± 3.8
9	47.5	62.5	65	50	60	50	55	57.5	55.9 ± 6.4
10	62.5	62.5	65	55	55	55	57.5	55	58.4 ± 4.2
11	42.5	60	72.5	72.5	75	72.5	70	72.5	67.2 ± 11
12	50	45	42.5	57.5	65	65	65	65	56.9 ± 9.7
13	40	45	57.5	57.5	52.5	60	60	45	**52.2 ± 7.8**
14	52.5	60	55	55	50	60	45	55	**54.1 ± 5**
15	57.5	60	62.5	65	72.5	72.5	65	72.5	65.9 ± 6
16	50	55	62.5	65	72.5	72.5	65	72.5	64.4 ± 8.4
17	60	57.5	57.5	65	72.5	60	72.5	67.5	64.1 ± 6.3
18	45	45	55	65	67.5	67.5	65	67.5	59.7 ± 9.9
19	40	37.5	45	35	40	40	22.5	42.5	**37.8 ± 6.9**
20	52.5	55	55	47.5	60	62.5	65	62.5	57.5 ± 6
21	47.5	40	40	27.5	45	55	52.5	52.5	**45 ± 9**
22	42.5	35	52.5	52.5	67.5	55	55	65	**53.1 ± 10.7**
23	47.5	55	55	47.5	62.5	60	60	50	**54.7 ± 5.9**
24	35	35	22.5	35	42.5	42.5	37.5	50	**37.5 ± 8**
25	62.5	62.5	67.5	67.5	62.5	67.5	65	62.5	64.7 ± 2.5
26	55	62.5	57.5	62.5	57.5	57.5	55	55	57.8 ± 3.1

## References

[1] Langston J. W. (2002). Parkinson's disease: current and future challenges. *NeuroToxicology*.

[2] de Lau L. M., Breteler M. M. (2006). Epidemiology of Parkinson's disease. *The Lancet Neurology*.

[3] de Rijk M. C., Launer L. J., Berger K. (2000). Prevalence of Parkinson's disease in Europe: a collaborative study of population-based cohorts. *Neurology*.

[4] Jankovic J. (2007). Parkinson's disease: clinical features and diagnosis. *Journal of Neurology, Neurosurgery and Psychiatry*.

[5] Ho A. K., Iansek R., Marigliani C., Bradshaw J. L., Gates S. (1998). Speech impairment in a large sample of patients with Parkinson's disease. *Behavioural Neurology*.

[6] Logemann J. A., Fisher H. B., Boshes B., Blonsky E. R. (1978). Frequency and cooccurrence of vocal tract dysfunctions in the speech of a large sample of Parkinson patients. *Journal of Speech and Hearing Disorders*.

[7] Little M. A., McSharry P. E., Roberts S. J., Costello D. A. E., Moroz I. M. (2007). Exploiting nonlinear recurrence and fractal scaling properties for voice disorder detection. *BioMedical Engineering OnLine*.

[8] Little M. A., McSharry P. E., Hunter E. J., Spielman J., Ramig L. O. (2009). Suitability of dysphonia measurements for telemonitoring of Parkinson's disease. *IEEE Transactions on Biomedical Engineering*.

[9] Duffy J. (2012). *Motor Speech Disorders: Substrates, Differential Diagnosis, and Management*.

[10] McNeil M. (2008). *Clinical Management of Sensorimotor Speech Disorders*.

[11] Baken R. J., Orlikoff R. F. (1999). *Clinical Measurement of Speech and Voice*.

[12] Dejonckere P. H., Bradley P., Clemente P. (2001). A basic protocol for functional assessment of voice pathology, especially for investigating the efficacy of (phonosurgical) treatments and evaluating new assessment techniques: guideline elaborated by the Committee on Phoniatrics of the European Laryngological Society (ELS). *European Archives of Oto-Rhino-Laryngology*.

[13] Sakar B. E., Isenkul M. E., Sakar C. O. (2013). Collection and analysis of a Parkinson speech dataset with multiple types of sound recordings. *IEEE Journal of Biomedical and Health Informatics*.

[14] Tsanas A., Little M. A., McSharry P. E., Spielman J., Ramig L. O. (2012). Novel speech signal processing algorithms for high-accuracy classification of Parkinsons disease. *IEEE Transactions on Biomedical Engineering*.

[15] Tsanas A., Little M. A., McSharry P. E., Ramig L. O. (2010). Accurate telemonitoring of Parkinson’s disease progression by noninvasive speech tests. *IEEE Transactions on Biomedical Engineering*.

[16] Sakar C. O., Kursun O. (2010). Telediagnosis of parkinson's disease using measurements of dysphonia. *Journal of Medical Systems*.

[17] Shahbaba B., Neal R. (2009). Nonlinear models using dirichlet process mixtures. *Journal of Machine Learning Research*.

[18] Das R. (2010). A comparison of multiple classification methods for diagnosis of Parkinson disease. *Expert Systems with Applications*.

[19] Guo P.-F., Bhattacharya P., Kharma N. (2010). Advances in detecting Parkinson’s disease. *Medical Biometrics*.

[20] Luukka P. (2011). Feature selection using fuzzy entropy measures with similarity classifier. *Expert Systems with Applications*.

[21] Li D.-C., Liu C.-W., Hu S. C. (2011). A fuzzy-based data transformation for feature extraction to increase classification performance with small medical data sets. *Artificial Intelligence in Medicine*.

[22] Ozcift A., Gulten A. (2011). Classifier ensemble construction with rotation forest to improve medical diagnosis performance of machine learning algorithms. *Computer Methods and Programs in Biomedicine*.

[23] Åström F., Koker R. (2011). A parallel neural network approach to prediction of Parkinson's Disease. *Expert Systems with Applications*.

[24] Polat K. (2012). Classification of Parkinson's disease using feature weighting method on the basis of fuzzy C-means clustering. *International Journal of Systems Science*.

[25] Chen H.-L., Huang C.-C., Yu X.-G. (2013). An efficient diagnosis system for detection of Parkinson's disease using fuzzy k-nearest neighbor approach. *Expert Systems with Applications*.

[26] Zuo W.-L., Wang Z.-Y., Liu T., Chen H.-L. (2013). Effective detection of Parkinson's disease using an adaptive fuzzy k-nearest neighbor approach. *Biomedical Signal Processing and Control*.

[27] Kursun O., Gumus E., Sertbas A., Favorov O. V. (2012). Selection of vocal features for Parkinson's Disease diagnosis. *International Journal of Data Mining and Bioinformatics*.

[28] Bhattacharya I., Bhatia M. SVM classification to distinguish Parkinson disease patients.

[29] Ramig L. O., Sapir S., Fox C., Countryman S. (2001). Changes in vocal loudness following intensive voice treatment (LSVT®) in individuals with Parkinson's disease: a comparison with untreated patients and normal age-matched controls. *Movement Disorders*.

[30] Gibbons J. (2010). *Nonparametric Statistical Inference*.

[31] Hollander M., Wolfe D. A. (2013). *Nonparametric Statistical Methods*.

[32] Kendall M. M. (1990). *Rank Correlation Methods*.

